# Convergent evolution of antibiotic resistance mechanisms between pyrrolobenzodiazepines and albicidin in multidrug resistant *Klebsiella pneumoniae*

**DOI:** 10.1038/s44259-025-00104-4

**Published:** 2025-06-06

**Authors:** Yasmin M. Surani, Matthew E. Wand, Pietro Picconi, Michele Di Palma, Riccardo Zenezini Chiozzi, Md. Mahbub Hasan, Paolo Andriollo, Stefan Grätz, Kazi S. Nahar, Michael Maynard-Smith, Roderich D. Süssmuth, Roberto A. Steiner, Khondaker Miraz Rahman, Charlotte K. Hind, J. Mark Sutton

**Affiliations:** 1 UK Health Security Agency, Research and Evaluation, Porton Down, Salisbury, SP4 0JG Wiltshire UK; 2 Institute of Pharmaceutical Science, School of Cancer & Pharmaceutical Sciences, King’s College London, Franklin-Wilkins Building, 150 Stamford Street, London, SE1 9NH UK; 3 Department of Biomedical Sciences, University of Padova, Viale G. Colombo 3, 35131 Padova, Italy; 4 UCL Mass Spectrometry Science Technology Platform, Division of Biosciences, Darwin Building, University College London, Gower Street, London, WC1E 6BT UK; 5 Department of Genetic Engineering and Biotechnology, Faculty of Biological Sciences, University of Chittagong, Chattogram, 4331 Bangladesh; 6 Department of Chemistry, Technische Universität Berlin, Straße des 17. Juni 124, 10623 Berlin, Germany; 7 Randall Centre for Cell and Molecular Biophysics, King’s College London, Guy’s Campus, London, SE1 1UL UK

**Keywords:** Drug discovery, Evolution, Genetics, Microbiology, Molecular biology, Structural biology

## Abstract

Pyrrolobenzodiazepines (PBDs) containing C8-linked aliphatic heterocycles have been developed as a new class of potent antibacterial compounds. They are active against multidrug resistant Gram-negative pathogens, including *Klebsiella pneumoniae*. When isolates were exposed to PBDs, they acquired resistance, with significant increases in inhibitory concentrations. Resistant strains showed mutations in genes associated with resistance to albicidin, specifically *tsx* and *merR*-family regulator *albA*. Heterologous expression of AlbA in *E. coli* and introducing the L120Q AlbA resistance-mediating modification into the genome of a sensitive *K. pneumoniae* strain conferred PBD and albicidin resistance. Proteomic analysis of the resistant strains showed elevated AlbA protein levels compared to isogenic wild-type strains. Crystallographic studies with the antibiotic binding domain of AlbA show binding of KMR-14-14 to the same groove shown to bind albicidin. Given the parallels between these two structurally unrelated compound classes, AlbA may offer resistance to further antibiotics and should be considered in future antibiotic discovery.

## Introduction

Antibiotics are heavily relied upon in the clinic, both to treat and prevent bacterial infection^1^. Exposure to antibiotics presents a survival challenge to the bacteria, which may result in the emergence of resistant populations through genomic mutations followed by either vertical or horizontal transmission^2^. Multidrug-resistant (MDR) bacterial populations are on the rise, and pose a real threat to public health, both economically and with regards to the loss of human life^1^.

Species of particular concern have been termed the ESKAPE pathogens: *Enterococcus faecium*, *Staphylococcus aureus*, *Klebsiella pneumoniae*, *Acinetobacter baumannii*, *Pseudomonas aeruginosa*, and *Enterobacter species*^3^. Of these, the last four are Gram-negative bacteria, which present a greater challenge due to the need to penetrate their two cell membranes with very different properties while avoiding drug efflux^4,5^.

*K. pneumoniae* inhabits the gastrointestinal tract, but it is also an opportunistic hospital-associated pathogen. One-third of all Gram-negative infections are attributed to it, including pneumonia, urinary tract infections, wound colonisation, and, more seriously: endocarditis, and septicaemia. *K. pneumoniae* strains have exhibited resistance to the four major antibiotic classes: third generation cephalosporins, aminoglycosides, fluoroquinolones, and even carbapenems, which were a last resort against extended-spectrum β-lactamase (ESBL) producers^2^.

Pyrrolobenzodiazepines (PBDs) are sequence-selective DNA minor groove binding agents with antitumour^6^ and antibacterial^7^ activities. While their antitumour activities have been shown to be due to covalent DNA binding and transcription factor inhibition^8^, their antibacterial activities are thought to be due to a combination of DNA binding and the inhibition of DNA gyrase^9^. The core PBD scaffold has been described in *Streptomyces* species^10^, naturally-occurring PBDs have also been identified in *Klebsiella* spp^11^, and synthetic derivatives have demonstrated activity against Gram-negative bacteria including *K. pneumoniae*^9^. These compounds represent a potential new scaffold for antibiotic development. The antibacterial PBDs have been designed to simultaneously minimise eukaryotic toxicity whilst incorporating chemical features thought to facilitate Gram-negative entry ^9,12^.

Resistance to PBDs in *K. pneumoniae* has been observed through sequence changes in a number of genes, including those encoding Tsx and MerR-family regulator AlbA^9^. Tsx is an outer membrane nucleoside transporter^13^, and AlbA is a transcriptional regulator capable of binding antibiotics^14^. Both of these proteins are associated with albicidin, a DNA gyrase-targeting natural product from *Xanthomonas albilineans*^15^. The former is the mechanism by which albicidin enters the cell, and both this and the latter gene have been implicated as mechanisms of albicidin resistance in *Klebsiella* species^13,16^.

This study seeks to validate the emerging parallels between PBDs and albicidin. These are two sets of antibacterial compounds of disparate but bacterial origin, with the same molecular target, found to have the same mechanism of cell entry, and now potentially to be subject to the same resistance mechanisms. Four lead compounds were assessed for activity and resistance, to draw conclusions about whether proteins of the AlbA family are indeed responsible for sequestering antibiotics of the PBD class.

The findings in this study have significant implications for new drug discovery, given the impact of this conserved mechanism of resistance between antimicrobial classes, especially given the diversity of the chemical structures. Two additional PBDs have been synthesised in this study to understand the significance of the resistance mechanism on a range of active compounds. Additional evidence has been generated around the role of AlbA and its drug binding domain (termed AlbAS) in mediating resistance, using model systems, proteomics, and crystallography.

## Results

### The lead PBD compounds are active against multidrug resistant Gram-negative pathogens

**Fig. 1:**
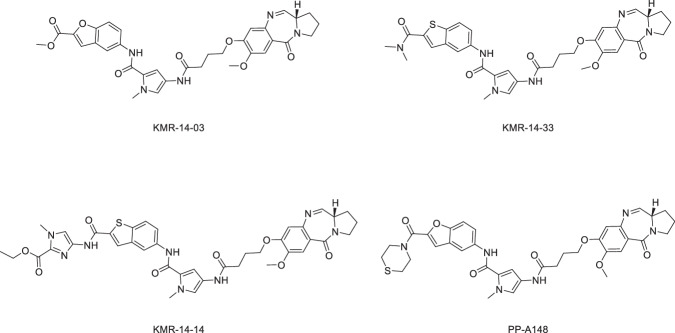
The chemical structures of the PBD compounds used in this study. The four lead compounds selected for this study were KMR-14-03, KMR-14-14,KMR-14-33, and PP-A148. Compounds KMR-14-33 and PPA148 were synthesised previously^9^ and labelled as 8 and 7 in the previous publication respectively. The syntheses of KMR-14-03 and KMR-14-14 are reported here for the first time.

We have previously described a series of PBD molecules with activity against a range of multi-drug resistant (MDR) Gram-negative and Gram-positive pathogens^9^; the synthesis and activity of two additional PBDs KMR-14-03 and KMR-14-14 is reported here. The PBD compounds, exemplified by KMR-14-03, KMR-14-14, KMR-14-33, and PP-A148 (Fig. 1), display promising activity against a range of Gram-negative pathogens (Table 1). Of particular note are the low MIC values against multidrug resistant *Klebsiella pneumoniae* and *Acinetobacter baumannii* strains, both priority ESKAPE pathogens, with the only exception PP-A148 against *K. pneumoniae* NCTC 13368. We did not see activity against the two isolates of *Pseudomonas aeruginosa* tested, up to a maximum concentration of 32 µg/mL. KMR-14-03 and KMR-14-33 showed good activity against all the *Burkholderia* species isolates tested, but no activity was seen at 32 µg/mL for KMR-14-14 with two strains: CEP509 and NCTC 10743, or with PP-A148 against C1962. Differences in the MIC values for specific compounds and in certain species or strains may reflect either influx limitations or efflux liabilities in these species, related to specific structural features.

**Table 1:** MIC data for the lead PBD compounds KMR-14-03, KMR-14-14, KMR-14-33, and PP-A148 against extended panels of MDR Gram-negative pathogens

Species	Strain	MIC (μg/mL)			
		KMR-14-03	KMR-14-14	KMR-14-33	PP-A148
*K. pneumoniae*	NCTC 13368	8	1	2	32
	M6	1	0.125	1	0.25–0.5
	NCTC 13438	1	0.25	0.5	4
	NCTC 13439	4	0.25–0.5	2	4-8
	NCTC 13443	4	1-2	2	16
	KP16	0.5	0.25–0.5	1	1
	NCTC 46704	4-8	0.25–0.5	1	4
	NCTC 51851	8	0.5	2	4
	MGH 78578	1-2	0.25	1	4–8
*A. baumannii*	NCTC 17978	1	1	0.5	2
	AYE	1	2	1	4
	NCTC 13424	1	1	0.5	2
	ADP1	0.25	0.25	0.125	1
	NCTC 13302	0.25	0.5	0.25	4
	UKA2	0.25	1	0.5	4
	UKA7	2	1	0.5	4
	W1	1	1	1	4
*P. aeruginosa*	PA01	>32	>32	>32	>32
	NCTC 13437	>32	>32	>32	>32
*B. multivorans*	C1576	4	2	1	16
	C1962	4	2	2	>32
*B. cenocepa*	K56-2	4	1	2	1
*B. cepacia*	CEP509	8	>32	2	8
	ATCC 17765	0.5	0.25	0.125	1
	LMG 17997	0.25	0.12	0.125	0.25
	NCTC 10743	8	>32	1-2	16

KMR-14-33 (8) and PP-A148 (7) were assayed previously^9^ and results were within two-fold of the current findings.

Evidence that efflux liability may not be a significant contributor in some species is shown from data with well-characterised efflux pump inhibitors, including PAβN which does not show any effect on MICs against KMR-14-14, except in the case of two *A. baumannii* strains (Supplementary Table 1). KMR-14-33 showed more marked decreases in MIC in the presence of efflux pump inhibitors across *K. pneumoniae* and *P. aeruginosa* strains as well as in *A. baumannii*. In *P. aeruginosa* strain PA01, the MIC value decreased from >32 µg/mL to 2 µg/mL with the addition of PAβN and magnesium.

### Breakthrough resistance is observed in *Klebsiella pneumoniae*

**Fig. 2:**
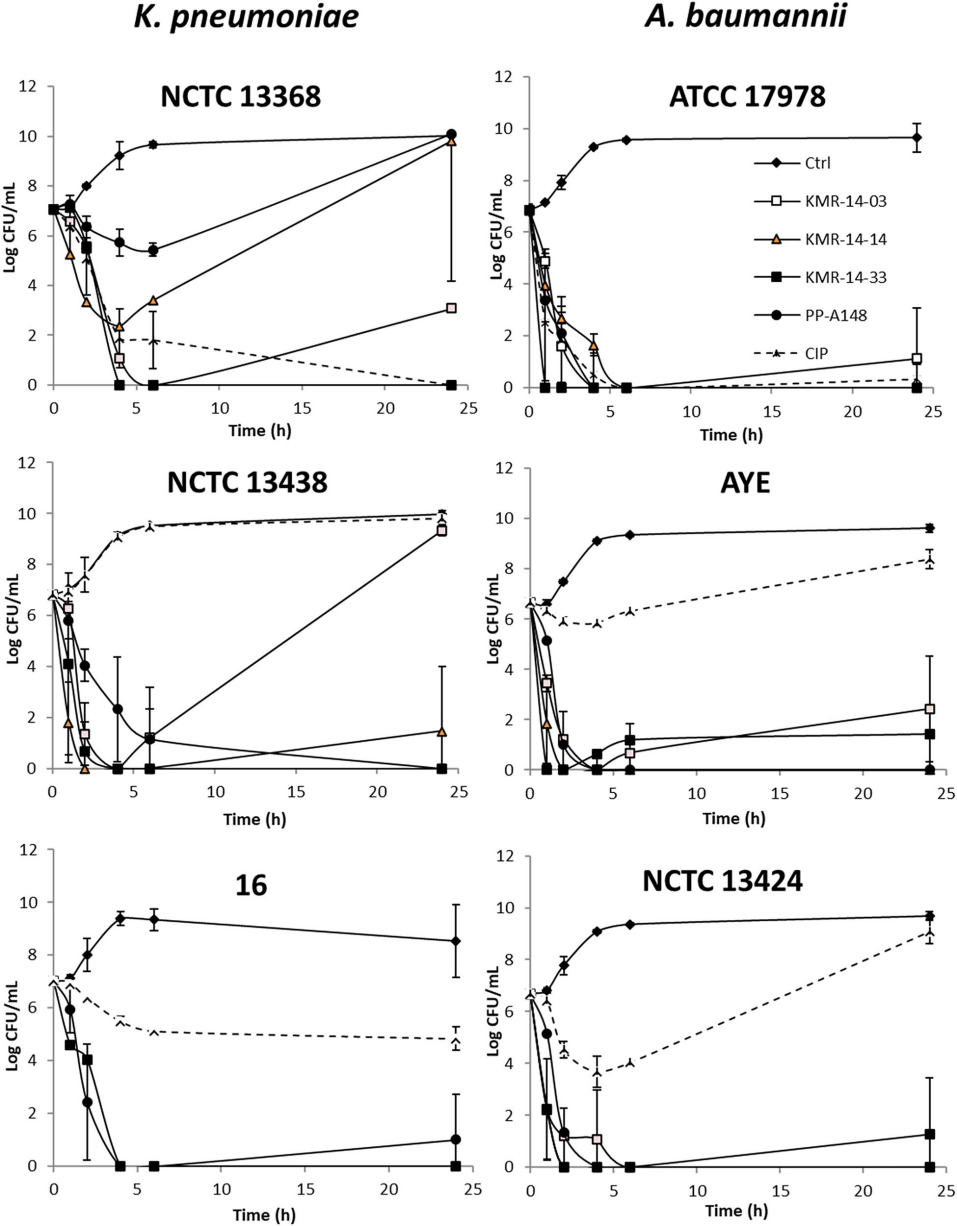
Time-kill data for the strains when subject to the PBD compounds or controls. Selected *K. pneumoniae* and *A. baumannii* strains were subject to 4x MIC concentrations of either one of the lead PBD compounds (KMR-14-03, KMR-14-14, KMR-14-33, or PP-A148) or ciprofloxacin. Data for compounds KMR-14-33 and PP-A148 were reported previously^9^.

When exposed to a concentration four times the MIC, increased resistance emerged to KMR-14-14 in NCTC 13368, a multidrug resistant strain of *K. pneumoniae* (Fig. 2), and similar results were previously observed for other PBDs in this strain background^7^. No breakthrough resistance was observed for PBDs in *A. baumannii* strains tested. Breakthrough resistance to 4x MIC was observed with the control antibiotic ciprofloxacin, another DNA gyrase inhibitor, in *A. baumannii* AYE and NCTC 13424, and *K. pneumoniae* NCTC 13438 and 16.

### Mutations in *tsx* contribute to PBD-resistance in *Klebsiella pneumoniae*

The genome sequences of the PBD-resistant isolates were determined. Mutations in the nucleoside transporter gene *tsx* were observed (Supplementary Table 2), with a modification resulting in a premature stop codon, or a deletion causing a frameshift and the production of a truncated protein likely to be inactive.

To confirm that mutations in *tsx* caused the observed increase in resistance to PBDs, compounds were evaluated in a *K. pneumoniae* strain containing a transposon inserted into its *tsx* gene: MKP103 Tn:*tsx*^17^. The MIC was substantially increased, between >2 and >8 fold relative to the wild-type strain (Table 2).

**Table 2:** MIC data for the lead PBD compounds KMR-14-03, KMR-14-14, KMR-14-33, and PP-A148 against the wildtype *K. pneumoniae* MKP103 strain and a MKP103 strain containing a transposon insertion in its *tsx* gene

	MIC (μg/mL)			
Strain	KMR-14-03	KMR-14-14	KMR-14-33	PP-A148
MKP103 WT	16	4	8–16	32
MKP103 Tn:*tsx*	>32	>32	>32	128

The resistant isolates were tested against KMR-14-14 in the presence of efflux pump inhibitors and membrane permeabilising agents to understand more about their entry and extrusion from the cell. Addition of a membrane permeabiliser, PMBN, to the *tsx* mutants consistently restored their susceptibility to the PBD compound, with MICs reduced by >8 to >16-fold (Table 3). This was not observed in the parental strain, demonstrating the effects of the reduced uptake in the *tsx* mutant strains. The RND-family efflux pump inhibitor, PAβN, also restored susceptibility, with a > 2 to >16-fold reduction in MIC. No effect on MIC was observed with CCCP, a general uncoupler of proton motive force.

**Table 3:** MIC data for KMR-14-14 against wild-type *K. pneumoniae* NCTC 13368 and KMR-14-14 resistant isolates in the presence of membrane-interactive agents

*CCCP* carbonyl cyanide *m*-chlorophenyl hydrazone, an uncoupler of the proton motive force. *PAβN* phenylalanine-arginine β-naphthylamide, an efflux pump inhibitor. *NMP* 1-(1-naphthylmethyl)-piperazine, another efflux pump inhibitor. *PMBN* polymyxin B nonapeptide (PMBN), an outer membrane permeabiliser. Shading indicates a significant result, defined as >2-fold change in MIC.

The resistant isolates did not display significant resistance to other established antibiotics, including representatives of fluoroquinolone, carbapenem, cephalosporin, macrolide, glycopeptide, and aminoglycoside classes (Supplementary Table 3).

### Mutations in *merR* contribute to PBD-resistance in *Klebsiella pneumoniae*

Mutations in a gene encoding a MerR-family protein (H50N and L120Q) were also identified in the whole genome sequencing of PBD-resistant isolates of NCTC 13368 and NCTC 13438 respectively (Supplementary Table 4). These mutations featured alongside SNPs in other genes: a phosphonate transporter in NCTC 13368 and a transcription regulator and a cation efflux system in NCTC 13438. The specific MerR-family protein was identified as AlbA, a protein that has previously been associated with resistance to albicidin^18^.

To evaluate the significance of an *albA* single-nucleotide modification independently of the other mutations, and thus the importance of *albA* in mediating resistance to PBDs, the L120Q modification was engineered into the genome of a drug sensitive *K. pneumoniae* strain: NCTC 7427, using an adapted recombineering method^19^. In the engineered strain, the presence of the modification gave rise to a 32-fold increase in MIC relative to the parent strain (Table 4).

**Table 4:** MIC data for the lead PBD compounds KMR-14-03, KMR-14-14, KMR-14-33, and PP-A148 against wild-type *K. pneumoniae* NCTC 7427 and a strain engineered to contain the L120Q modification in the *albA* gene

Strain	MIC (μg/mL)			
	KMR-14-03	KMR-14-14	KMR-14-33	PP-A148
NCTC 7427	0.125–0.25	0.0625	0.125–0.25	0.25
NCTC 7427 AlbA L120Q	4	2	4	>4

**Fig. 3:**
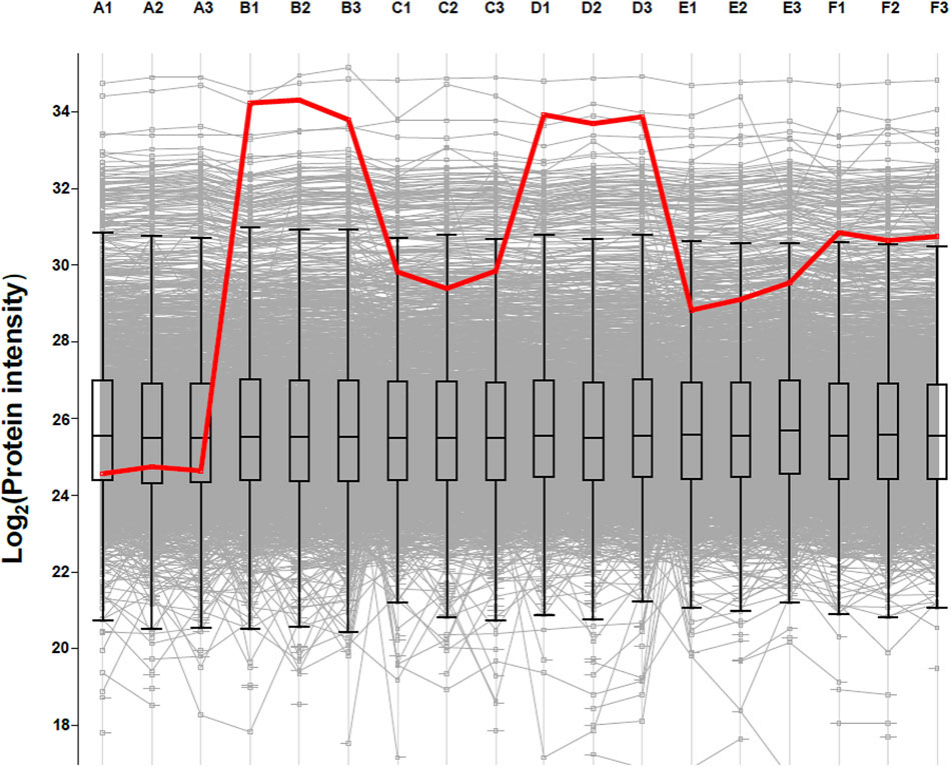
Proteomics data for engineered and adapted *K. pneumoniae* strains illustrating relative levels of AlbA protein. The engineered NCTC 7427 AlbA L120Q strain (**B**), PBD-adapted NCTC 13438 and NCTC 13368 strains with L120Q (**D**) and H50N (**F**) modifications respectively, are shown alongside parental strains (**A**, **C**, **E**). AlbA protein levels are indicated in red; other cellular proteins are shown in grey with error bars denoting average expression levels for cellular proteins in general. Numbers 1–3 show three replicate samples.

To explore the functional significance of the nucleotide modifications, proteomics was performed to look for changes in protein expression level. The engineered *K. pneumoniae* NCTC 7427 strain and the PBD-adapted strains with *albA* mutations, together with the corresponding wild-type strains, were treated with one-quarter MIC of KMR-14-14, and total cellular protein harvested. In each case, the strains with either L120Q or H50N SNPs showed increased AlbA expression relative to the parental strain (Fig. 3).

### Antimicrobial PBDs share resistance mechanisms with albicidin

It is intriguing that the synthetic PBDs generated in this study share two mechanisms of resistance with the naturally occurring antibiotic albicidin, and both PBDs and albicidin are indicated as DNA gyrase inhibitors^9,18^. To determine whether the *albA* gene product could capture the PBD compounds in a similar manner to albicidin, AlbA was expressed in *Escherichia coli*. The DNA sequence for the drug-binding domain of the *K. pneumoniae* protein, termed AlbAS (or TipAS), was cloned into an IPTG-inducible expression vector pET28a(+) and used to transform a high expressing *E. coli* strain BL21(DE3).

**Fig. 4:**
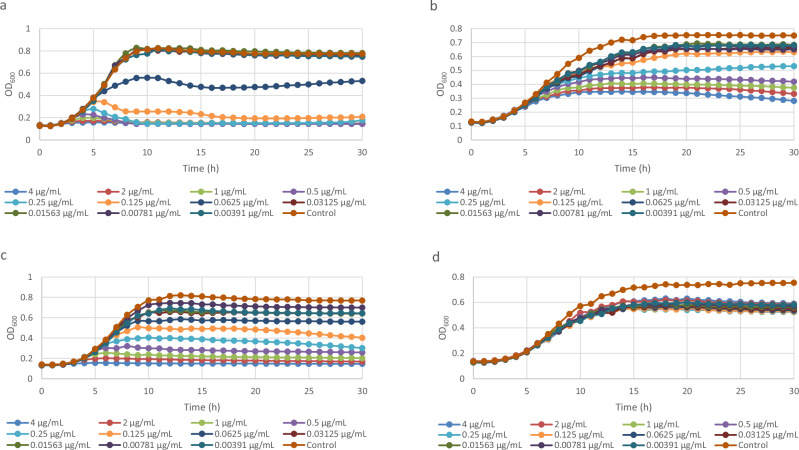
Inducible expression of the *K. pneumoniae* AlbAS protein in *E. coli* mediates resistance to PBDs and albicidin. BL21 DE3 *E. coli* carrying inducible AlbAS-expressing pET28a(+) plasmid, was grown for 30 h at 25 °C and OD_600_ monitored hourly, in the presence of **a**) KMR-14-14, **b**) KMR-14-14 + IPTG, **c**) albicidin, and **d**) albicidin + IPTG. The antimicrobials were serially diluted 2-fold, and IPTG used at a fixed concentration of 0.2 mM to estimate an MIC with and without AlbAS expression. Results represent the average of three biological replicates.

The cells were treated with either a PBD compound or albicidin, and either with or without IPTG induction, and the growth monitored for 30 h at a temperature that favours soluble expression of AlbAS (25 °C). With all compounds, there was a marked increase in the basal concentration at which the cells were able to grow in the presence of IPTG (Table 5). Based on the threshold used for MIC determinations (endpoint OD_600_ > 0.1), we estimated that the MIC equivalent doses, where no growth is detected, were 0.25 and 2 µg/mL for KMR-14-14 and albicidin respectively, increasing to >4 µg/mL for both compounds when AlbAS expression was induced with IPTG (Fig. 4). This indicates that expression of the AlbAS protein confers albicidin and PBD resistance to otherwise susceptible *E. coli* strains. The lower overall OD_600_ values recorded for IPTG-induced bacteria (Fig. 4 panel B and D) may reflect the metabolic cost of overexpressing this resistance protein in the recombinant host cell.

**Table 5:** MIC data for the lead PBD compounds KMR-14-03, KMR-14-14, KMR-14-33, and PP-A148 and albicidin against *E. coli* strain BL21 DE3 containing the *K. pneumoniae albAS* gene on an IPTG-inducible pET28a(+) vector

Strain	MIC (μg/mL)				
	Albicidin	KMR-14-03	KMR-14-14	KMR-14-33	PP-A148
BL21 DE3 AlbAS uninduced	2	1-2	0.125–0.5	1–4	2
BL21 DE3 AlbAS induced	>4	>4	>4	>4	>4

### Structural basis of the AlbAS interaction with KMR-14-14

**Fig. 5:**
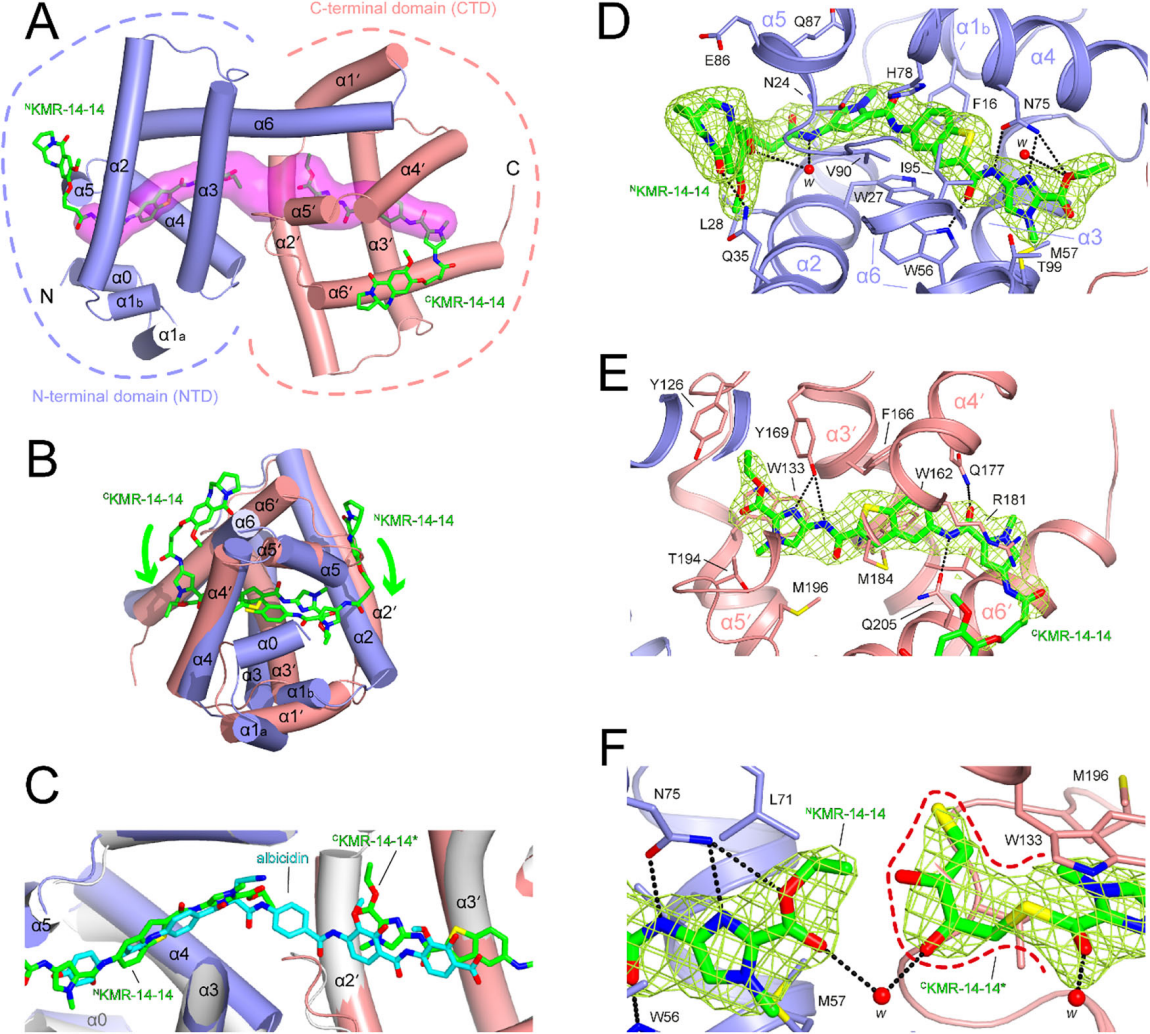
X-ray structure of AlbAS in complex with KMR-14-14. **A** Overall structure of AlbAS with its all-helical N-terminal (NTD, α0-α6) and C-terminal (CTD, α1’-α6’) domains shown in blue and salmon respectively. A 40 Å central tunnel^44^, represented as a semi-transparent surface in magenta, runs through the structure and hosts two KMR-14-14 molecules. These, shown in green in stick representation, bind within each domain (^N^KMR-14-14 and ^C^KMR-14-14 bound to NTD and CTD respectively) with their ester ‘tails’ pointing toward the middle of the tunnel whilst their PBD ‘heads’ protrude outside. **B** Superposition of NTD and CTD highlighting their structural similarity. KMR-14-14 molecules bind in opposite direction with the common protein frame. **C** Superposition of AlbAS:KMR-14-14 and AlbAS:albicidin complexes (PDB 6ET8). NTD and CTD of the AlbAS:KMR-14-14 complex are shown with the usual colour code whilst AlbAS of the AlbAS:albicidin complex is shown in grey. A single albicidin molecule (shown in cyan) binds within the central tunnel across both NTD and CTD domains with one of its *p*-aminobenzoic acid groups roughly positioned in the gap between the ester tails of ^N^KMR-14-14 and ^C^KMR-14-14. Interactions of ^N^KMR-14-14 (**D**) and ^C^KMR-14-14 (**E**) within their hosting domains. 2mFo-DFc electron density for the ligands is shown at the 1.0 σ level in light green. Residues within 3.8 Å of the ligand are labelled and shown as stick representation, colour-coded according to the domain to which they belong. Hydrogen bonds are shown as black dashed lines. The red spheres labelled ‘*w*’ represent water molecules. **F** Reaction between ^C^KMR-14-14 and DTT gives a thioester derivative (^C^KMR-14-14*, highlighted by the red broken line).

To understand how PBDs can interact with the drug-binding domain of AlbA we have solved the X-ray structure of *Klebsiella oxytoca* AlbAS in complex with KMR-14-14 at 2.17 Å resolution. Data collection and refinement statistics are available in Supplementary Table 5. The AlbAS protein (residues 1-221, corresponding to residues 128–348 of full-length AlbA) is an all-helical architecture formed by two structurally similar N-terminal and C-terminal domains (NTD residues 1-115 and CTD residues 116–221, respectively) that share low sequence identity (15.6%). NTD and CTD are arranged in tandem following a pseudo two-fold rotation, giving rise to a 40 Å-long central tunnel in which two KMR-14-14 molecules are bound (Fig. 5A).

The KMR-14-14 ligands are arranged in a ‘tail-to-tail’ fashion such that their ester ends face each other at the AlbAS core whilst their PBD ‘heads’ protrude outside the tunnel bending toward the protein’s molecular surface thanks to their flexible -C_3_H_6_- linker. This arrangement is conserved in all three independent AlbAS molecules in the asymmetric unit. As both ligands point their ends toward the centre of the tunnel this implies that the similar NTD and CTD frames that are tandemly organized stabilise KMR-14-14 in opposite directions (Fig. 5B).

The binding stoichiometry observed here in which NTD and CTD each stabilise a KMR-14-14 ligand differs from what was previously seen with albicidin^16,18^. The latter straddles across both domains following the slightly bent central tunnel (Fig. 5C). Thus, whilst albicidin binds to AlbAS with a 1:1 ratio, KMR-14-14 binds with a 2:1 stoichiometry or, in other words, with a 1:1 ratio with respect to each domain.

Electron density for the tunnel-buried tails is well defined for all KMR-14-14 ligands (Fig. 5D, E). Differently, electron density for the solvent exposed PBD moieties is clearly visible only for one NTD-bound ligand thanks to the additional stabilisation of a neighbouring AlbAS molecule within the crystal (Fig. 5D and Supplementary Fig. 1), while it is of poorer quality for the others (Fig. 5E and Supplementary Fig. 2).

A combination of hydrophobic interactions and hydrogen bonds involving residues that are typically conserved stabilise KMR-14-14 ligands within each domain. At the NTD, key H-bond contributors are W56 and N75. More specifically, the side chain of the latter is engaged in multiple interactions with the amide, *N*-methyl-imidazole, and ester groups at the ligand’s tail whilst the indole group of W56 is H-bonded to the carbonyl oxygen next to the thiophene ring (Fig. 5D). The latter interaction forces a different rotamer for W56 compared to that seen in the complex with albicidin (Supplementary Fig. 3).

A few solvent-mediated interactions further stabilise the ligand at the NTD. At the CTD, H-bonds are instead observed between Y169 and the nitrogen atoms of the ligand’s *N*-methyl-imidazole and its flanking amide, as well as between Q177 and Q205 and the amide next to the *N*-methyl-pyrrole moiety (Fig. 5E). Additionally, a π-π stacking interaction is established between the indole side chain of W133 and ligand’s *N*-methyl-imidazole. Overall, NTD and CTD stabilise KMR-14-14 in opposite directions via domain-specific interactions in keeping with their low sequence similarity despite their common overall architecture.

### KMR-14-14 undergoes chemical modification in the AlbAS tunnel

As noted earlier, two KMR-14-14 molecules insert their ester tails in the tunnel leaving a gap in the middle (Fig. 5A). In the albicidin complex^16,18^ this region is occupied by one of the two *p*-aminobenzoic acid moieties of the ligand (Fig. 5B). During crystallographic refinement we noticed excess electron density at the ester tail in two out of three of the CTD-bound KMR-14-14 ligands that suggested that a chemical modification might have occurred (Supplementary Fig. 4).

We could confidently model such additional density considering the formation of a thioester derivative following a reaction between the ester group and DTT present in the crystallisation mixture (Fig. 5F). This hypothesis is reinforced by the presence of a DTT molecule in the gap between the two ester tails in the only AlbAS molecule with unreacted KMR-14-14 ligands (Supplementary Fig. 5).

This serendipitous observation strengthens the idea that, as with albicidin, the central portion of the tunnel tends to be naturally filled with molecular fragments with a somewhat hydrophobic character. Taken together, our structural results indicate the tail that emanates from the C8 atom of the PBD moiety is a critical determinant for the interaction with AlbAS. Therefore, ligand optimisation focused on this region holds great promise for the development of PBDs able to evade AlbA resistance.

## Discussion

C8-linked pyrrolobenzodiazepine monomers (PBDs) have demonstrated promising activity as potent antimicrobial drugs against both Gram-positive and Gram-negative bacteria. The PBD compounds evaluated in this study are of interest as potential clinical antibiotics due to their potency against a series of multidrug resistant Gram-negative pathogens, including *Klebsiella pneumoniae*, *Acinetobacter baumannii*, and *Burkholderia* species.

Some *K. pneumoniae* isolates were observed to develop elevated levels of resistance to the PBD compounds when exposed to four times the minimum inhibitory concentration (MIC) for 24 h in time kill experiments. Of the genomic changes found in resistant isolates, an unexpected observation was point mutations in the *albA* gene^9^. The *albA* gene has been reported to confer resistance to albicidin in *Klebsiella oxytoca*^18^. The coincidence of both mechanism of action and mechanisms of resistance, between PBDs and the phytotoxin albicidin, was intriguing and merited more detailed studies. This study validates the observation that the AlbA protein can indeed protect Gram-negative bacteria from both the assayed PBD compounds and albicidin. This knowledge may be useful in the design of future antibiotics based on these scaffolds, or other DNA gyrase targeting agents^20^.

Given the differing origin of these two known AlbA substrates, and their lack of structural similarity, the possibility that this may be a relevant resistance mechanism for other antibiotics should be considered. Structural information obtained here from AlbA’s ligand-binding domain in complex with KMR-14-14 suggests that other long hydrophobic molecules of the PBD class can be efficiently sequestered within its tunnel. However, their exact binding pose will likely be determined by molecule-specific interactions. Moreover, the different binding stoichiometry between KMR-14-14 and albicidin, with the former binding to each contributing AlbAS domain, allows us to hypothesise that PBDs could be employed to evaluate their specific effect on the mechanism of transcription activation that is currently incompletely understood.

As well as being almost ubiquitous in *Klebsiella* species isolates, a homologous gene to *albA* is present in many other Gram-negative species, albeit not at the same prevalence. These include *Pseudomonas aeruginosa*, *Burkholderia spp*, *Acinetobacter baumannii*, and many other species within the *Enterobacteriaceae*. Although not described here, overexpression of the AlbA homolog in these other species would be expected to mediate resistance to PBD compounds too. A related gene is associated with resistance to a wide range of antimicrobial agents, sharing features with thiostreptone, in Gram-positive bacteria^21^. Given the observations in this study, where cross resistance is evident to two distinct gyrase inhibitor classes, a re-evaluation of the importance of this family of antibiotic-sequestering protein is merited. An improved understanding of the structure-function relationships, both in terms of drug specificity and the functional significance of the genes in other bacteria, would enable the community to understand if these resistance determinants are of concern for new antibiotics and facilitate efforts to evade their effects by synthetic chemistry modifications.

## Methods

### Chemical synthesis of KMR-14-03 and KMR-14-14

#### General chemistry

Reagents and solvents were purchased from Sigma Aldrich, with building blocks from Fluorochem or Activate Scientific. All reactions were monitored by thin layer chromatography using Merck 60 F254 silica gel coated plates; visualisation was achieved using UV light at 254 nm. Flash column chromatography was carried out using silica gel (60 Å pore size, 230–400 mesh, 43–60 µm particle size), supplied by Merck.

Nuclear magnetic resonance spectra were recorded at 400 MHz on a Bruker Avance III HD NanoBay (Ascend™ magnet). All chemical shifts are quoted in ppm relative to internal resonance. The multiplicity of the signal is designated by the following abbreviations: s = singlet, d = doublet, t = triplet, q = quartet, m = multiplet, and br refers to a broad signal. The coupling constants are recorded in Hz. Analytical LC-MS was performed using an Agilent Technologies 1290 Infinity II system, equipped with a Phenomenex Onyx Monolithic C18 column (50 × 4.6 mm). The King’s College London Mass Spectrometry Facility undertook HRMS ESI-MS analysis.

Solvents were evaporated using a KNF RC-600 system with a SC-920G pump. Compounds were further dried using either a Schlenk line attached to an Edwards RV5 pump (high vacuum line) or a Thermo Scientific Vacutherm vacuum oven at 40 °C with a KNF pump. Glassware was dried in a Memmert UN55 oven at 200 °C. Compounds were weighed using Sartorius balances, accurate to at least four significant figures.

#### Synthesis of methyl 5-(4-((tert-butoxycarbonyl)amino)-1-methyl-1H-pyrrole-2-carboxamido)benzo[b]thiophene-2-carboxylate (**1.3**)


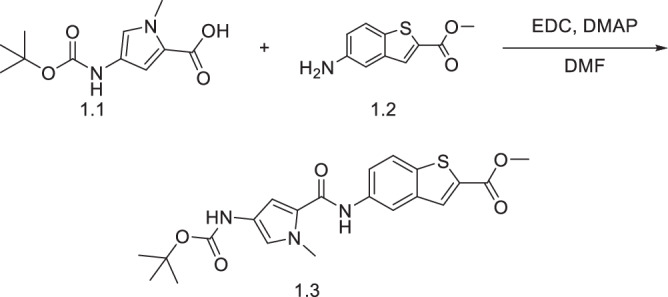


Acid **1.1** (150 mg, 0.624 mmol, 1.0 eq.) was dissolved in DMF (2.5 mL) and activated with EDC (2.0 eq, 239.37 mg, 1.25 mmol) and DMAP (2.5 eq, 190.69 mg, 1.56 mmol) for 30 min with stirring. Amine **1.2** (1.5 eq, 194.08 mg, 0.936 mmol) was added, and the mixture stirred overnight. The reaction was quenched with ice-cold water (50 mL) and extracted with ethyl acetate (3 × 50 mL). The combined organic fractions were washed sequentially with 1 M citric acid, saturated sodium bisulphate, water, and brine, dried over magnesium sulphate, filtered, and solvent evaporated. The crude was purified by flash column chromatography (95:5 dichloromethane:ethyl acetate) to give product **1.3** (214 mg, 79.8%). ^1^H NMR (400 MHz, DMSO-d_6_) *δ* 9.98 (s, 1H), 9.13 (br. s., 1H), 8.45 - 8.51 (m, 1H), 8.17 (s, 1H), 7.97 (d, *J* = 8.80 Hz, 1H), 7.79 (dd, *J* = 1.83, 8.80 Hz, 1H), 6.97 (d, *J* = 8.99 Hz, 2H), 3.89 (s, 3H), 3.83 (s, 3H), 1.46 (s, 9H). ^13^C NMR (101 MHz, DMSO-d_6_) *δ* 162.4, 159.8, 152.9, 138.8, 137.0, 135.8, 133.1, 131.0, 122.8, 122.5, 122.4, 121.5, 115.9, 78.3, 52.6, 36.2, 28.2. [M + H]^+^ LCMS C_21_H_24_N_3_O_5_S calculated. 430.50 found 430.1.

#### Synthesis of 5-(4-((tert-butoxycarbonyl)amino)-1-methyl-1H-pyrrole-2-carboxamido)benzo[b]thiophene-2-carboxylic acid (**1.4**)


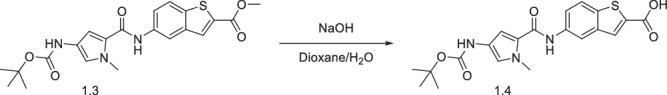


Compound **1.3** (78 mg, 0.18 mmol, 1 eq.) was dissolved in dioxane (2 mL) and sodium hydroxide (72.64 mg, 1.82 mmol; 10 eq.) dissolved in water (1 mL) was added dropwise. After 2 h, the dioxane was evaporated, and the residue dissolved in water (20 mL) and acidified by the dropwise addition of 1 M HCl to pH 2.0. The crude was extracted using ethyl acetate (3 x 30 mL), the combined organic fractions dried over magnesium sulphate and filtered, and the solvent evaporated to give compound **1.4** (42 mg, 56%). ^1^H NMR (400 MHz, DMSO-d_6_) *δ* 9.96 (s, 1H), 9.13 (s, 1H), 8.44 (d, *J* = 1.83 Hz, 1H), 8.05 (s, 1H), 7.94 (d, *J* = 8.80 Hz, 1H), 7.76 (dd, *J* = 2.02, 8.80 Hz, 1H), 6.97 (d, *J* = 6.24 Hz, 2H), 3.83 (s, 3H), 1.46 (s, 9H). ^13^C NMR (101 MHz, DMSO-d_6_) *δ* 163.6, 159.9, 152.9, 139.1, 136.9, 135.9, 130.2, 122.8, 122.6, 122.5, 121.1, 115.8, 104.8, 48.6, 40.2, 40.0, 39.8, 39.6, 36.2, 28.2. [M + H]^+^ LCMS C_20_H_22_N_3_O_5_S calculated. 416.47 found 416.1.

#### Synthesis of ethyl 4-(5-(4-amino-1-methyl-1H-pyrrole-2-carboxamido)benzo[b]thiophene-2-carboxamido)-1-methyl-1H-imidazole-2-carboxylate (**1.5**)


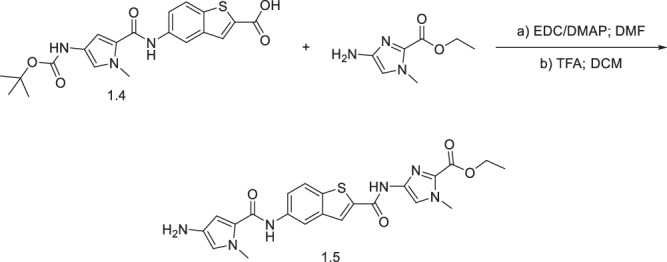


Compound **1.4** (40 mg, 0.096 mmol, 1.0 eq.) was dissolved in DMF (2.5 mL) and activated with EDC (2.0 eq, 36.91 mg, 0.193 mmol) and DMAP (2.5 eq, 29.41 mg, 0.241 mmol) for 30 min with stirring. Ethyl 4-amino-1-methyl-1H-imidazole-2-carboxylate (1.5 eq, 24.43 mg, 0.144 mmol) was added, and the mixture stirred overnight. The reaction was quenched with ice-cold water (50 mL), extracted with ethyl acetate (3 × 50 mL), dried over magnesium sulphate, filtered, and solvent evaporated. The crude product was dissolved in DCM (2.5 mL) and TFA (1 mL) was added. The mixture was stirred for 1 h, solvent evaporated, and purified by flash column chromatography (95:5 ethyl acetate:methanol) to give product **1.5** (18 mg, 55%). ^1^H NMR (400 MHz, DMSO-d_6_) *δ* 11.52 (s, 1H), 9.69 (s, 1H), 8.37 (s, 2H), 7.92 (d, *J* = 8.80 Hz, 1H), 7.67–7.78 (m, 2H), 6.52 (d, *J* = 2.02 Hz, 1H), 6.36 (d, *J* = 1.83 Hz, 1H), 4.30 (q, *J* = 7.15 Hz, 2H), 3.96 (s, 3H), 3.76 (s, 3H), 1.33 (t, *J* = 6.42 Hz, 3H). ^13^C NMR (101 MHz, DMSO-d_6_) *δ* 159.2, 158.5, 139.7, 137.2, 137.1, 134.9, 131.8, 122.6, 116.1, 115.9, 115.8, 104.2, 41.6, 35.8, 35.6, 31.6, 29.4, 22.1, 14.1. [M + H]^+^ LCMS C_22_H_23_N_6_O_4_S calculated. 467.53 found 467.1.

#### Synthesis of ethyl (S)-4-(5-(4-(4-((7-methoxy-5-oxo-2,3,5,11a-tetrahydro-1H-benzo[e]pyrrolo[1,2-a][1,4]diazepin-8-yl)oxy)butanamido)-1-methyl-1H-pyrrole-2-carboxamido)benzo[b]thiophene-2-carboxamido)-1-methyl-1H-imidazole-2-carboxylate (**KMR-14-14**)


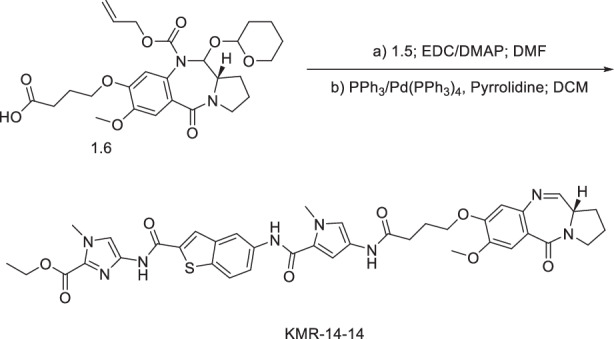


The PBD core **1.6** was synthesised using a nine-step literature procedure^22^. **1.6** (22 mg, 0.042 mmol, 1.1 eq.) was dissolved in DMF (2.5 mL) and activated with EDC (2.0 eq, 16.27 mg, 0.0849 mmol) and DMAP (2.5 eq, 12.96 mg, 0.106 mmol) for 30 min with stirring. Compound **1.5** (1.0 eq, 18 mg, 0.040 mmol) was added, and the mixture stirred overnight. The reaction was quenched with ice-cold water (50 mL), extracted with ethyl acetate (3 × 50 mL), dried over magnesium sulphate, filtered, and solvent evaporated. The crude was dissolved in DCM (2.5 mL), and Pd(PPh_3_)_4_ (0.05 eq, 2.45 mg, 2.12 µmol), PPh_3_ (0.25 eq, 2.78 mg, 10.6 µmol), and pyrrolidine (1.5 eq, 5.22 µL, 63.6 µmol), were added sequentially. After 30 min, the solvent was evaporated, and the crude purified by flash column chromatography (93:7 ethyl acetate:methanol) to give **KMR-14-14** (7.5 mg, 23%). ^1^H NMR (400 MHz, DMSO-d_6_) *δ* 11.54 (s, 1H), 9.98 (s, 1H), 9.94 (s, 1H), 9.04 (s, 1H), 8.39 (s, 2H), 7.94 (d, *J* = 8.80 Hz, 1H), 7.74 - 7.80 (m, 1H), 7.72 (s, 1H), 7.34 (s, 1H), 7.24 (br. s., 1H), 7.13 (s, 1H), 7.03 (s, 1H), 6.99 (dd, *J* = 1.93, 8.34 Hz, 1H), 6.65 - 6.74 (m, 1H), 4.30 (d, *J* = 6.97 Hz, 2H), 3.97 (s, 3H), 3.86 (s, 3H), 3.83 (s, 3H), 3.67 (s, 1H), 2.73 (s, 1H), 2.45 -2.54 (m, 2H), 1.85 - 2.13 (m, 6H), 1.29 - 1.32 (m, 3H). ^13^C NMR (101 MHz, DMSO-d_6_) *δ* 172.5, 168.9, 159.8, 158.4, 153.3, 150.2, 146.9, 140.2, 139.8, 139.6, 137.2, 134.1, 131.3, 124.1, 123.1, 122.7, 115.6, 60.7, 55.6, 36.2, 35.5, 34.4, 34.4, 33.8, 33.2, 31.5, 31.3, 30.4, 29.4, 29.0, 28.8, 28.7, 28.2, 23.7, 22.1, 14.1, 13.9. [M + H]^+^ HRMS C_39_H_41_N_8_O_8_S calculated. 781.27626 found 781.2768.

#### Synthesis of methyl 5-((4-((tert-butoxycarbonyl)amino)-1-methyl-1H-pyrrol-2-yl)carbamoyl)benzofuran-2-carboxylate (**2.2**)


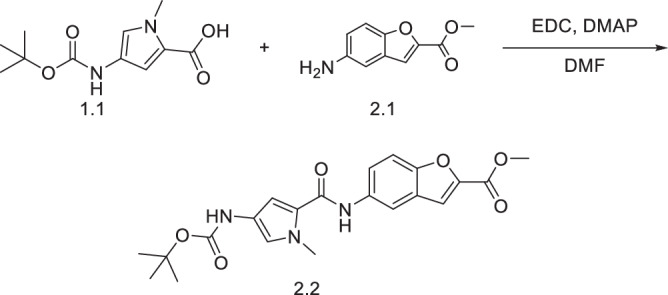


Acid **1.1** (0.50 g, 2.1 mmol, 1.0 eq) was dissolved in DMF (3 mL). EDC (2.0 eq, 0.65 g, 4.2 mmol) and DMAP (2.5 eq, 0.64 g, 5.2 mmol) were added, and the solution stirred for 30 min. Methyl 5-aminobenzofuran-2-carboxylate (2.0 eq, 0.80 g, 4.2 mmol) was added and the reaction mixture stirred overnight. The reaction was quenched in ice-cold water (30 mL) and extracted with ethyl acetate (3 × 50 mL). The organic layers were combined and washed with 1 M citric acid solution (50 mL), sodium bicarbonate solution (50 mL), water (50 mL) and brine (50 mL). The solution was concentrated in a rotary evaporator to yield a brown oil. The crude was purified using flash column chromatography (80:20 dichloromethane:ethyl acetate) to produce the protected benzofuran-pyrrole side chain **2.2** in 72% yield as a brown solid. ^1^H NMR (400 MHz, CDCl_3_) δ 8.11 (d, 1H, *J* = 2.0 Hz), 7.65 (s, 1H), 7.54 (d, 1H, *J* = 8.8 Hz), 7.50 (d, 1H, *J* = 0.8 Hz), 7.38 (dd, 1H, J = 2.0, 8.8 Hz), 6.86 (s, 1H), 6.68 (s, 1H), 3.98 (s, 3H), 3.93 (s, 3H), 1.52 (s, 9H).). ^13^C NMR (101 MHz, CDCl_3_) *δ* 159.9, 159.7, 152.5, 146.2, 134.0, 127.4, 123.3, 121.9, 121.3, 118.8, 114.2, 113.8, 112.6, 103.9, 80.4, 52.5, 36.8, 28.4. [M + H] + EIMS C21H23N3O6 calculated 414.4 found 415.0.

#### Synthesis of methyl 5-((4-amino-1-methyl-1H-pyrrol-2-yl)carbamoyl)benzofuran-2-carboxylate (**2.3**)


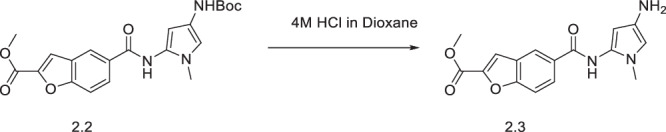


The Boc protected building block **2.2** was dissolved in methanol (1 mL for every 1 mg of starting material and 4 M HCl in dioxane (2 mL for every 25 mg of starting material) was added dropwise. Gas evolution was observed. After 1 h the solution was concentrated using a rotary evaporator to yield the deprotected compound **2.3** as the hydrochloric acid salt in 92% yield. ^1^H NMR (400 MHz, DMSO-d_6_) δ 10.14 (s, 1H), 10.04 (bs, 2H), 7.82 (s, 1H), 7.81 (s, 1H), 7.79-7.71 (m, 2H), 7.20 (d, 1H, *J* = 2.0 Hz), 7.18 (d, 1H, *J* = 2.0 Hz), 3.91 (s, 3H), 3.90 (s, 3H). [M + H]^+^ EIMS C_16_H_15_N_3_O_4_ calculated 313.3 found 313.9.

#### (S)-methyl 5-(4-(4-((7-methoxy-5-oxo-2,3,5,11a-tetrahydro-1H-benzo[e]pyrrolo[1,2-a][1,4]diazepin-8-yl)oxy)butanamido)-1-methyl-1H-pyrrole-2-carboxamido)benzofuran-2-carboxylate (**KMR-14-03**)


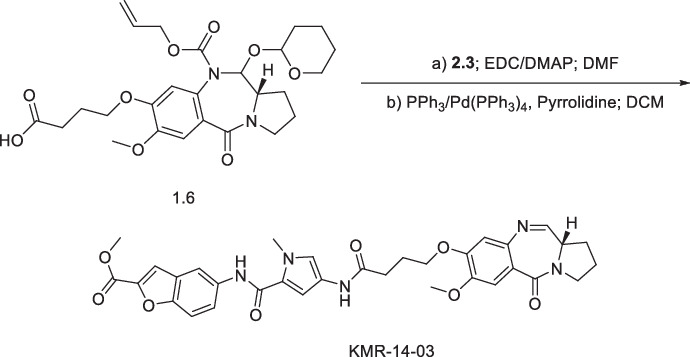


**1.6** (132 mg, 0.254 mmol, 1.1 eq.) was dissolved in DMF (3 mL) and activated with EDC (2.0 eq, 85 mg, 0.44 mmol) and DMAP (2.5 eq, 71 mg, 0.58 mmol) for 30 min with stirring. Compound **2.3** (1.0 eq, 70 mg, 0.23 mmol) was added, and the mixture stirred overnight. The reaction was quenched with ice-cold water (50 mL), extracted with ethyl acetate (3 × 50 mL), dried over magnesium sulphate, washed with 1 M citric acid, sodium bicarbonate solution, brine, filtered, and solvent evaporated. The crude was dissolved in DCM (10 mL), and Pd(PPh_3_)_4_ (0.05 eq, 3.54 mg, 3.06 µmol), PPh_3_ (0.25 eq, 3.99 mg, 15 µmol), and pyrrolidine (1.5 eq, 6.45 µL, 78.6 µmol) were added sequentially. After 30 min, the solvent was evaporated, and the crude purified by flash column chromatography (75:25 DCM:acetone) to give **KMR-14-03** in 64% yield as creamy white solid. ^1^H NMR (400 MHz, CDCl_3_) *δ* 8.40 (s, 1H), 8.17 (s, 1H), 8.12 (s, 1H), 7.65 (d, 1H, *J* = 4.4 Hz), 7.49-7.47 (m, 3H), 7.44 (s, 1H), 7.12 (d, 1H, *J* = 1.6 Hz) 6.80 (s, 1H), 6.33 (d, 1H, *J* = 1.6 Hz), 4.07 (t, 2H, *J* = 6 Hz) 3.97 (s, 3H), 3.87 (s, 3H), 3.86-3.75 (s, 3H), 3.74-3.69 (m, 2H) 3.59-3.52 (m, 1H) 2.33 (t, 2H, *J* = 6.0 Hz), 2.33-2.26 (m, 2H), 2.23-2.18 (m, 2H) 2.08-1.98 (m, 2H). ^13^C NMR (101 MHz, CDCl_3_) *δ* 170.0, 164.6, 162.8, 160.0, 152.4, 150.7, 147.7, 146.0, 140.6, 134.5, 127.2, 123.0, 121.6, 120.4, 119.9, 114.2, 112.3, 111.7,110.9, 104.3, 69.5, 68.1, 56.1, 53.8, 52.4, 46.7, 36.8, 32.9, 31.8, 29.5, 29.3, 24.9, 24.2. [M + H]^+^ HRMS C_33_H_33_N_5_O_8_ calculated. 628.2402 found 628.2397.

### Minimum inhibitory concentration (MIC) assays

MICs were determined using the broth microdilution method, as described by CLSI^23^. Compounds were solubilised in dimethyl sulfoxide (DMSO) prior to concentration adjustment in tryptic soy broth (TSB) media. The MIC was defined as the lowest concentration of compound which resulted in no visible growth at an optical density of 600 nm (OD_600_) after incubation at 37 °C for 20–24 h. Experiments were performed in triplicate. Optical density readings were taken on a BMG Labtech CLARIOstar microplate reader, a no bacteria control was used for blank subtraction, and DMSO alone with concentrations below 2% v/v was used to control for solubilised compounds.

### Minimum inhibitory concentration (MIC) assays with efflux pump inhibitors (EPIs) or membrane permeabilisers

An adapted microbroth dilution method was performed to evaluate either the efflux potential or extrusion of the compounds. 50 μL/well of EPI or membrane permeabiliser at four times final concentration and 50 μL/well of bacterial culture at OD_600_ 0.02 were added to wells containing 100 μL/well of a dilution series of compound. Carbonyl cyanide *m*-chlorophenyl hydrazone (CCCP) was used at a final concentration of 10 µg/mL, 1-(1-naphthylmethyl)-piperazine (NMP) at 50 µg/mL, polymyxin B nonapeptide (PMBN) at 30 µg/mL, or phenylalanine-arginine β-naphthylamide (PAβN) at 25 µg/mL. With PaβN, media was supplemented with magnesium sulphate (MgSO_4_) (40 μM) to prevent permeabilisation of the outer membrane of Gram-negative bacteria^24^. A decrease in MIC of the compound of greater than two-fold was deemed significant. Experiments were performed in triplicate.

### Time kill assays

For each compound TSB media (3 mL) in a glass bijou was inoculated with an overnight culture of bacteria to a final concentration of OD_600_ 0.02 of bacterial culture. Bacteria were challenged with four times the MIC concentration of each compound and incubated at 37 °C in a rotary shaker at 180 rpm. 100 µL aliquots were taken at 0, 1, 2-, 4-, 6-, and 24-hours post-inoculation and serial dilutions performed in sterile water. Total viable counts were determined by the Miles Misra dilution method^25^. The compound is deemed bactericidal if the bacterial titre was reduced by greater than 3 log_10_ CFU/mL, and bacteriostatic if the titre was decreased by 0 − 3 log_10_ CFU/mL. Experiments were performed in triplicate.

### Engineering SNPs into *Klebsiella pneumoniae* NCTC 7427

The L120Q SNP was engineered into the *K. pneumoniae* NCTC 7427 *albA* gene (NCBI accession number UGLP01000001, region 1725088-1726134, locus tag 01643) using an adapted version of a protocol outlined in the literature^19^.

The pORTMAGE311B plasmid was provided in an *E. coli* host strain, DNA was isolated via miniprep. To electroporate pORTMAGE311B into NCTC 7427 to allow gene editing to take place, cells were first made electrocompetent. 500 µL starter culture was added to 50 mL Luria Bertani (LB) media and grown at 37 °C with 180 rpm shaking until OD_600_ 0.3–0.5. The cells were cooled on ice for ten minutes, then pelleted by centrifugation at 4700 rpm for ten minutes at 4 °C. The pellet was successively washed with 10 mL, 5 mL, and 1 mL ice-cold 300 mM sucrose, each time resuspended by gentle pipetting, and centrifuged at 3000 g for three minutes at 4 °C. The electrocompetent cells were then resuspended in 200 µL ice-cold 300 mM sucrose, and 40 µL was combined with 1 µL plasmid DNA (100 ng/µL) in a pre-chilled 1 mm gap cuvette on ice. The cells were electroporated at 1.8 kV, immediately resuspended in 950 µL room temperature Super Optimal broth with Catabolite repression (SOC) media and incubated at 37 °C for one hour with shaking at 180 rpm. The cells were plated on LB agar containing 50 µg/mL kanamycin, and grown overnight at 37 °C.

The *albA*-targeting oligonucleotide to generate the L120Q modification was designed with the help of the Mage Oligo Design Tool (MODEST)^26^ (Supplementary Fig. 6). *K. pneumoniae* NCTC 7427 carrying pORTMAGE311B had to be made competent to accept an *albA*-targeting oligonucleotide. The single-stranded DNA binding protein (Beta, from the λ red system) and dominant negative mismatch repair allele (MutL E32K) also needed to be induced with *m*-toluic acid.

Starter culture was added to 50 mL LB media containing 50 µg/mL kanamycin at an initial concentration of OD_600_ 0.1 and grown at 37 °C with 180 rpm shaking until OD_600_ 0.3–0.5. 50 µL 1 M *m*-toluic acid in ethanol was added and shaking continued for 30 min. The cells were cooled on ice for 15 min, then pelleted by centrifugation at 4700 rpm for ten minutes at 4 °C. As before, the pellet was successively washed with 10 mL, 5 mL, and 1 mL ice-cold 300 mM sucrose, each time resuspended by gentle pipetting, and centrifuged at 3000 *g* for three minutes at 4 °C. The electrocompetent cells were then resuspended in 200 µL ice-cold 300 mM sucrose, and 40 µL was combined with 1 µL 100 µM oligonucleotide in a pre-chilled 2 mm gap cuvette on ice. The cells were electroporated at 2.5 kV, immediately resuspended in 1 mL room temperature SOC media, combined with 4 mL Terrific Broth (TB) media, and incubated at 37 °C for one hour with 180 rpm shaking. 5 mL LB media and 10 µL 50 mg/mL kanamycin was added, and incubation continued for 24 hours. The cells were plated on LB agar containing 0.125 µg/mL KMR-14-14, and grown overnight at 37 °C.

### Whole genome sequencing

NCTC 13368 and NCTC 13438 PBD-resistant isolates and NCTC 7427 AlbA L120Q were analysed to identify and confirm mutations. DNA was isolated using the Promega Wizard® Genomic DNA Purification Kit and the genome was sequenced by the UKHSA-GSDU (UK Health Security Agency Genomic Services and Development Unit) on a HiSeq 2500 (Illumina) with paired-end read lengths of 150 bp. DNA libraries were prepared using the Nextera DNA Flex library prep kit according to manufacturer’s instructions. FastQ reads were quality trimmed using Trimmomatic^27^ and draft chromosomes scaffolds were assembled using SPAdes 3.15.3^28^ where contigs of less than 1 kb were filtered out. FastQ reads from exposed isolates were subsequently mapped to their respective wild-type pre-exposure chromosomal sequence using BWA 0.7.5^29^. BAM format files were generated using Samtools^30^ and VCF files constructed using GATK2 Unified Genotyper (version 0.0.7)^31^. These were further filtered using a filtering criteria of Mapping quality >30, Genotype quality >40, Variant ratio >0.9, Read depth >10 to identify high confidence mutations. All the above sequencing analysis was performed using UKHSA Galaxy. BAM files were visualised in Integrative Genomics Viewer (IGV version 2.3.55) (Broad Institute). Individual mutations were identified using Galaxy^32^ for the NCTC 13368 and NCTC 13438 strains. The presence of the L120Q AlbA modification in NCTC 7427 was confirmed using whole genome sequencing in a similar manner.

### Proteomics to evaluate AlbA expression level

Starter cultures were added to 7 mL TSB media at OD_600_ 0.1 and grown at 37 °C with 180 rpm shaking for one hour. The cultures were diluted to OD_600_ 0.25 in 4 mL TSB, one-quarter MIC KMR-14-14 was added and shaking at 37 °C was continued for 30 min. The cultures were harvested by centrifugation at 4500 rpm for 20 minutes, and the pellets resuspended in 1 mL lysis buffer: 5% SDS in 100 mM Tris pH 8.5. The samples were boiled at 95 °C for 10 min at 1200 rpm.

5 mM Tris(2-carboxyethyl)phosphine (TCEP) and 20 mM chloroacetamide (CAA) were added, and the samples were pulse-sonicated with a micro-tip for two minutes at 80% amplitude. Proteins were precipitated through the addition of four volumes of methanol, one volume of chloroform, and three volumes of distilled water. The solutions were vortexed, centrifuged at 10,000 *g* for five minutes, the supernatants were discarded, and the pellets air-dried.

The pellets were suspended in digestion buffer: 4% sodium deoxycholate (SDC) in 100 mM Tris pH 8.5, and trypsin was added in a 1:50 enzyme to protein ratio. The samples were left to digest at 37 °C 1200 rpm overnight, then the reaction stopped by adding trifluoroacetic acid (TFA) to pH 2, which precipitates SDC for removal. This process was carried out in a high throughput manner^33^ on a KingFisher Apex robot (Thermo Fisher Scientific).

Peptides were dissolved in 2% formic acid before liquid chromatography–tandem mass spectrometry (LC-MS/MS) analysis. The mixture of tryptic peptides was analysed using an Ultimate3000 high-performance liquid chromatography (UHPLC) system coupled online to an Eclipse mass spectrometer (Thermo Fisher Scientific). Buffer A consisted of water acidified with 0.1% formic acid; buffer B was 80% acetonitrile and 20% water with 0.1% formic acid. The peptides were first trapped for one minute at 30 μL/min with 100% buffer A on a trap (0.3 mm x 5 mm with PepMap C18, 5 μm, 100 Å; Thermo Fisher Scientific). After trapping, the peptides were separated by a 50 cm analytical column (Acclaim PepMap, 3 μm; Thermo Fisher Scientific). The gradient was 7-35% B in 44 min at 300 nL/min. Buffer B was then raised to 55% in two minutes and increased to 99% for the cleaning step. Peptides were ionised using a spray voltage of 2.1 kV and a capillary heated at 280 °C. The mass spectrometer was set to acquire full-scan MS spectra (350 to 1400 mass/charge ratio) for a maximum injection time set to Auto at a mass resolution of 60,000 and an automated gain control (AGC) target value of 100%. For a second the most intense precursor ions were selected for MS/MS. Higher energy collision dissociation (HCD) fragmentation was performed in the HCD cell, with the readout in the Orbitrap mass analyser at a resolution of 15,000 (isolation window of 1.4 Th) and an AGC target value of 200% with a maximum injection time set to Auto and a normalised collision energy of 30%. All raw files were analysed by MaxQuant v2.1.4, searching against the strain’s specific FASTA (downloaded from NCBI) and the automatic settings, only adding (N) deamidation as a possible modification. “ProteinGroups.txt” was then filtered for contaminants and the data analysis was done in Perseus v.2.0.7 and Origin2022. The NCBI accession numbers for the AlbA protein are STU18836, STS95960, and STT99297 from strains NCTC 7427, NCTC 13438, and NCTC 13368 respectively. Data are available via ProteomeXchange with identifier PXD059600.

### Generation of BL21 (DE3) strains expressing *Klebsiella oxytoca* and *Klebsiella pneumoniae albAS*

Codon-optimised (DNASTAR Lasergene 16) synthetic *K. oxytoca* and *K. pneumoniae albAS* (Supplementary Table 6) were cloned into pET28a(+) using Gibson assembly^34^ with the primer pairs listed (Supplementary Table 7). The plasmids were initially transformed into *E. coli* NEB-5-alpha, and, once DNA isolated (NEB Monarch® Plasmid Miniprep Kit) and sequence confirmed (MRC PPU DNA Sequencing and Services), into *E. coli* BL21 (DE3). Transformants were selected on LB agar containing 50 µg/mL kanamycin in each case.

### Growth curves with BL21 (DE3) strains expressing *Klebsiella pneumoniae albAS*

Each compound was assayed in the presence and absence of 0.2 mM isopropyl β-D-1-thiogalactopyranoside (IPTG) in water, which induces expression of the *K. pneumoniae albAS* gene on the pET28a(+) plasmid. The experiment without IPTG was setup exactly as the MIC experiment described above. The experiment with IPTG was setup in an analogous manner to the EPI MIC experiment. 50 μL/well of IPTG at four times final concentration and 50 μL/well of bacterial culture at OD_600_ 0.02 were added to wells containing 100 μL/well of a dilution series of compound. The plates were incubated at 25 °C for 30 h, and OD_600_ readings taken every hour. Experiments were performed in triplicate, and fold-increase in MIC in the presence of IPTG was reported.

### X-ray crystallographic studies of AlbAS in complex with KMR-14-14

The *E. coli* BL21(DE3) strain expressing *K. oxytoca albAS* was further processed for large-scale growth at 37 °C in TB medium supplemented with kanamycin (50 μg/mL) until an OD_600_ of 0.5-0.6. Temperature was then lowered to 18 °C and protein synthesis induced for about 18 h by the addition of 200 μM IPTG. The culture was centrifuged at 6000 *g* for 30 min at 4 °C and the cell pellet resuspended in buffer A (50 mM Tris-HCl, 200 mM NaCl, 20 mM imidazole, 1 mM DTT, pH 7.5) supplemented with a protease inhibitors cocktail (cOmplete™, Roche). Cell lysis was accomplished using a French press, processing the sample twice, and the insoluble material sedimented by centrifugation (35000 *g* for 30 min at 4 °C). The clarified lysate was filtered through a 0.45 μm membrane prior to loading it on a 5 mL HisTrap column (Cytiva) pre-equilibrated with buffer A for immobilised metal affinity chromatography (IMAC). The column was extensively washed with buffer A and proteins eluted with a gradient from 0% to 80% buffer B (50 mM Tris-HCl, 200 mM NaCl, 500 mM imidazole, 1 mM DTT, pH 7.5) over 15 column volumes. AlbAS-containing fractions were pooled, concentrated using a 10 kDa cut-off concentrator (Sartorius) for size-exclusion chromatography (SEC) using a Superdex 16/600 75 pg (Cytiva) column. The sample was eluted with storage buffer (50 mM Tris-HCl, 100 mM NaCl, 1 mM DTT, pH 7.5). The His6 purification tag was cleaved at 20 °C by overnight incubation of the eluted sample with TEV protease. The mixture was then loaded onto a pre-equilibrated HisTrap column and the untagged material present in the flow-through fraction was collected, concentrated, and further purified by SEC on a Superdex 16/600 75 pg (Cytiva) column equilibrated with storage buffer.

For co-crystallisation, AlbAS was concentrated to approximately 30 mg/mL and a 50 mM stock solution of KMR-14-14 in DMSO added to achieve mild stoichiometric excess. Ligand precipitation was immediately observed. After overnight incubation at 4 °C, the mixture was centrifuged at 13000 *g* for 30 min and the supernatant used for setting up crystallisation trials using the Oryx8 (Douglas Instruments) in two-drop MRC 96-well plates (Molecular Dimensions). Initial crystallisation experiments taking advantage of various commercial screens were performed using the vapour diffusion setup at 20 °C and a 1:1 protein:precipitant ratio in 600 nL sitting drops. Following optimisation, the best co-crystals of the AlbAS:KMR-14-14 complex grew using 1.2 M ammonium sulphate as a precipitant. For data collection, crystals were cryo-protected by the addition of 2.0 M sodium malonate and cryo-cooled in liquid nitrogen.

The best crystal afforded the measurement of a complete dataset at 2.17 Å resolution at the ID30B beamline of ESRF (Grenoble, France). Data were processed using XDS^35^ as implemented in the EDNA pipeline and the structure solved by the molecular replacement (MR) technique using the software package Phaser^36^ starting from ligand-free AlbAS (PDB code 6H95) as template. Model building and crystallographic refinement were performed using COOT^37^ and REFMAC5^38–40^, respectively, of the CCP4 suite^41^. Stereochemical restraints for the organic ligands were generated using Grade^42^. A summary of data collection and refinement statistics are shown in Supplementary Table 5. Structural images were prepared with PyMol (Schrödinger). Coordinates and structure factors for the AlbAS:KMR-14-14 complex have been deposited with the Protein Data Bank with accession code 8RKY.

## Supplementary information

Supplementary information

## Data Availability

Data is provided within the manuscript or supplementary information files. The crystallography data has been submitted to the Protein Data Bank with accession code 8RKY. The mass spectrometry proteomics data have been deposited to the ProteomeXchange Consortium via the PRIDE^43^ partner repository with the dataset identifier PXD059600.
