# Supplementary material for: Convergent evolution of antibiotic resistance mechanisms between pyrrolobenzodiazepines and albicidin in multidrug resistant Klebsiella pneumoniae
Source: NPJ Antimicrob Resist. 2025 Jun 6;3:52. doi: 10.1038/s44259-025-00104-4 (PMC12144233; doi:10.1038/s44259-025-00104-4)
Supplement: Supplementary file 1 — Supplementary information [file 44259_2025_104_MOESM1_ESM.pdf]

Supplementary material

Species	Strain	Name	MIC (μg/mL)		
			Compound	+CCCP	+PAβN + Mg
K. pneumoniae	M6	KMR-14-14	0.125	0.06	0.25
		KMR-14-33	1	0.125	0.25
	NCTC 13368	KMR-14-14	1	1	1
		KMR-14-33	2	2	0.5
	NCTC 13438	KMR-14-14	0.25	0.25	0.5
		KMR-14-33	0.5	0.5	0.25
A. baumannii	NCTC 17978	KMR-14-14	1	1	0.25
		KMR-14-33	0.5	0.25	0.25
	AYE	KMR-14-14	2	2	1
		KMR-14-33	1	0.125	0.25
	NCTC 13424	KMR-14-14	1	0.25	0.5
		KMR-14-33	0.5	0.25	0.25
P. aeruginosa	PA01	KMR-14-14	>32	>32	>32
		KMR-14-33	>32	>32	2
	NCTC 13437	KMR-14-14	>32	>32	>32
		KMR-14-33	>32	>32	16

Supplementary Table 1. MIC data for lead PBD compounds KMR-14-14 and KMR-14-33 against MDR Gram-negative pathogens in the presence of membrane-interactive agents. CCCP = carbonyl cyanide *m*-chlorophenyl hydrazone. PAβN = phenylalanine-arginine β-naphthylamide. Shading indicates a significant result.

Strain	Mutations	
	In *tsx*	Others
KP13368-14-14-R1	K122STOP in KPN_RS01945 (27% of reads)	
KP13368-14-14-R2	W34STOP in KPN_RS01945	Point mutation in formate dehydrogenase subunit alpha, N609I, KPN_RS10050; point mutation in glycosyltransferase family 4 protein, V29I; Del of nucleotide 89 in glycosyltransferase family 4 protein
KP13368-14-14-R3	Truncated 17aa protein in KPN_RS01945	Point mutation in 2-hydroxy-6-oxo-6-phenylhexa-2,4-dienoate hydrolase, N169K, KPN_RS11425

Supplementary Table 2. Whole genome sequencing data for *Klebsiella pneumoniae* NCTC 13368 with adaptations to KMR-14-14. The resistant isolates contain *tsx* mutations.

Strain	MIC (µg/mL)									
	14-14	14-33	CIP	LVX	MEM	FEP	AZM	TEC	GEN	AMK
KP13368 WT	1-4	2	0.5	1	0.25	32-64	64	>128	32	2-4
KP13368-14-14-R1	>32	8	0.5	1	0.25	32-128	32	>128	64	2-4
KP13368-14-14-R2	>32	8	0.5	1	0.25	32-128	32	>128	64	2-4
KP13368-14-14-R3	>32	8	0.5	1	0.25	32-64	32	>128	64	2-4

Supplementary Table 3. MIC data for established antibiotics against wild-type *Klebsiella pneumoniae* NCTC 13368 and KMR-14-14 resistant isolates. CIP = ciprofloxacin. LVX = levofloxacin. MEM = meropenem. FEP = cefepime. AZM = azithromycin. TEC = teicoplanin. GEN = gentamicin. AMK = amikacin.

Strain	Mutations	
	In *merR*	Others
KP13438-14-03-R2	L120Q in KPN_RS12075	Point mutation in *malT* transcriptional regulator, R268H, KPN_RS20465;<br>Point mutation in *silA*/*cusA* cation efflux system, Q384L
KP13368-148-R2	H50N in KPN_RS12075	Point mutation in phosphonate ABC transporter ATP-binding protein, KPN_RS01555

Supplementary Table 4. Whole genome sequencing data for two *Klebsiella pneumoniae* strains (NCTC 13438 and NCTC 13368) with adaptations to PBDs (KMR-14-03 and PP-A148 respectively). The PBD-resistant isolates both contain *merR* mutations.

Data collection

Data set	AlbAS:KMR-14-14
Beam Line	ID30B (ESRF)
Wavelength (Å)	0.9763
Resolution range[a] (Å)	49.49-2.17<br>(2.25-2.17)
Space group	*C*2
Cell dimensions<br>(*a*, *b*, *c*) (Å)<br>(α, β, γ) (°)	 182.63, 118.95, 57.70<br>90, 92.07, 90
Unique reflections[a]	64940<br>(6345)
Overall redundancy[a]	4.2<br>(4.4)
Completeness[a] (%)	99.8<br>(100.0)
$R_{merge}{}^{a}$ (%)	5.5<br>(143.5)
$R_{p.i.m.}$ (I)[a] (%)	3.0<br>(76.4)
CC(1/2)	0.999<br>(0.365)
$\langle I/\sigma(I) \rangle^{a}$	12.3<br>(0.9)

Refinement

PDB code	8RKY
R_{factor} (%)/R_{free}(%)	20.8/23.4
# non-H atoms	6088
protein	5492
water	226
ligands (KMR-14-14 and KMR-14-14*)	346
ligand (DTT)	24
Average *B* value (Å^2)	68.7
protein (chain A / chain B / chain C)	68.1/67.2/77.1
ligands (bound to chain A / chain B / chain C)	68.1/105.6/127.7
water	61.3
ligand (DTT)	119.8
rms bond lengths (Å)	0.005
rms bond angles (°)	1.27
Overall MolProbity score[b]	1.50 (98th percentile)

Supplementary Table 5. X-ray data collection and refinement statistics. [a] Numbers in parentheses refer to the highest resolution bin. *KMR-14-14 thioester. [b] MolProbity [45] score combines the clashscore, rotamer, and Ramachandran evaluations into a single score, normalised to be on the same scale as x-ray resolution. 100th percentile is the best among structures of comparable resolution; 0th percentile is the worst.

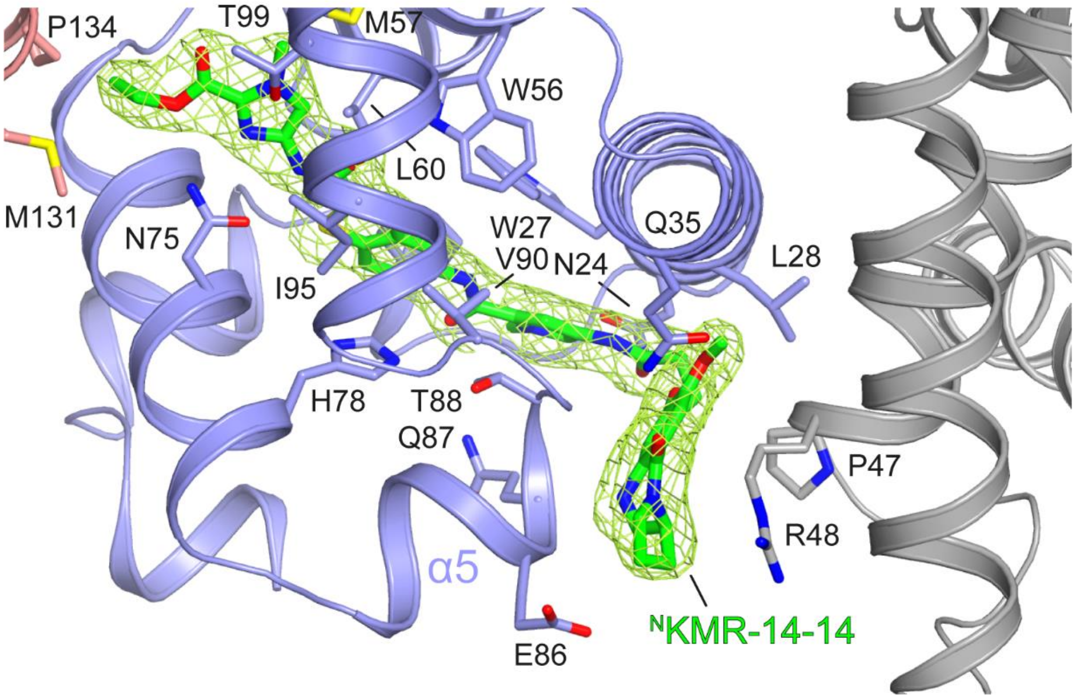

Supplementary Figure 1. Stabilisation of the PBD moiety. In one of the three AlbAS molecules present in the asymmetric unit, the PBD moiety bound to the NTD is stabilised by a neighbouring molecule (in grey). 2mFo-DFc electron density for the ligand is shown at the 1.0 σ level in light green. Residues within 3.8 Å of the ligand are labelled and shown as stick representation.

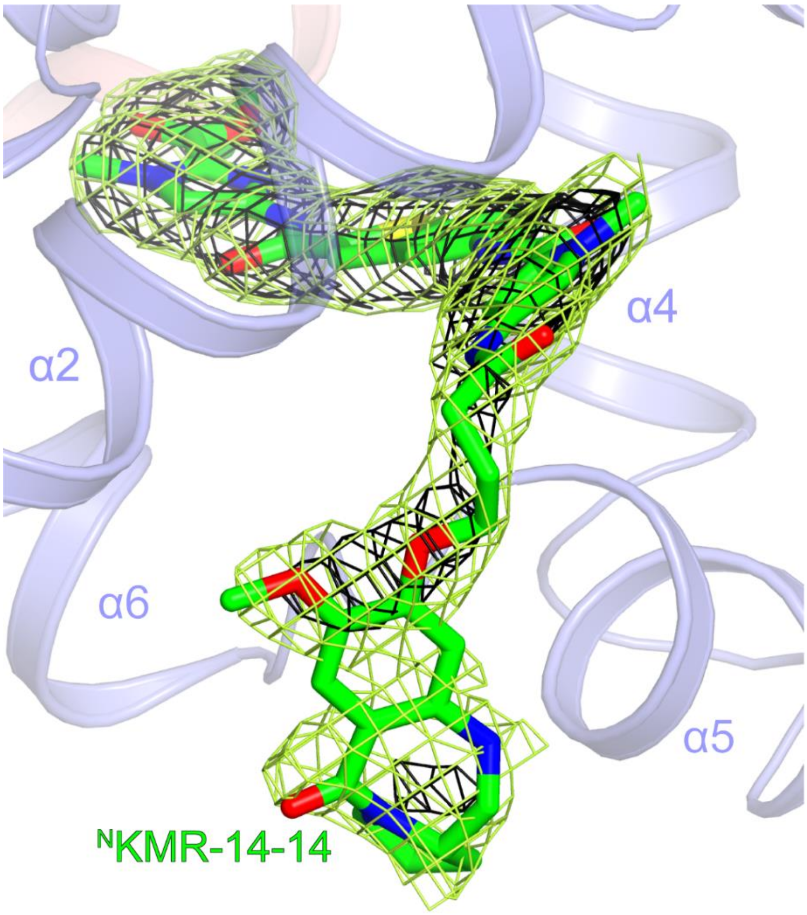

Supplementary Figure 2. Solvent-exposed KMR-14-14 PBD moieties are more flexible than tunnel-embedded tails. 2mFo-DFc electron density for the ligand is shown at the 0.5 σ and 1.0 σ levels in light green and black respectively.

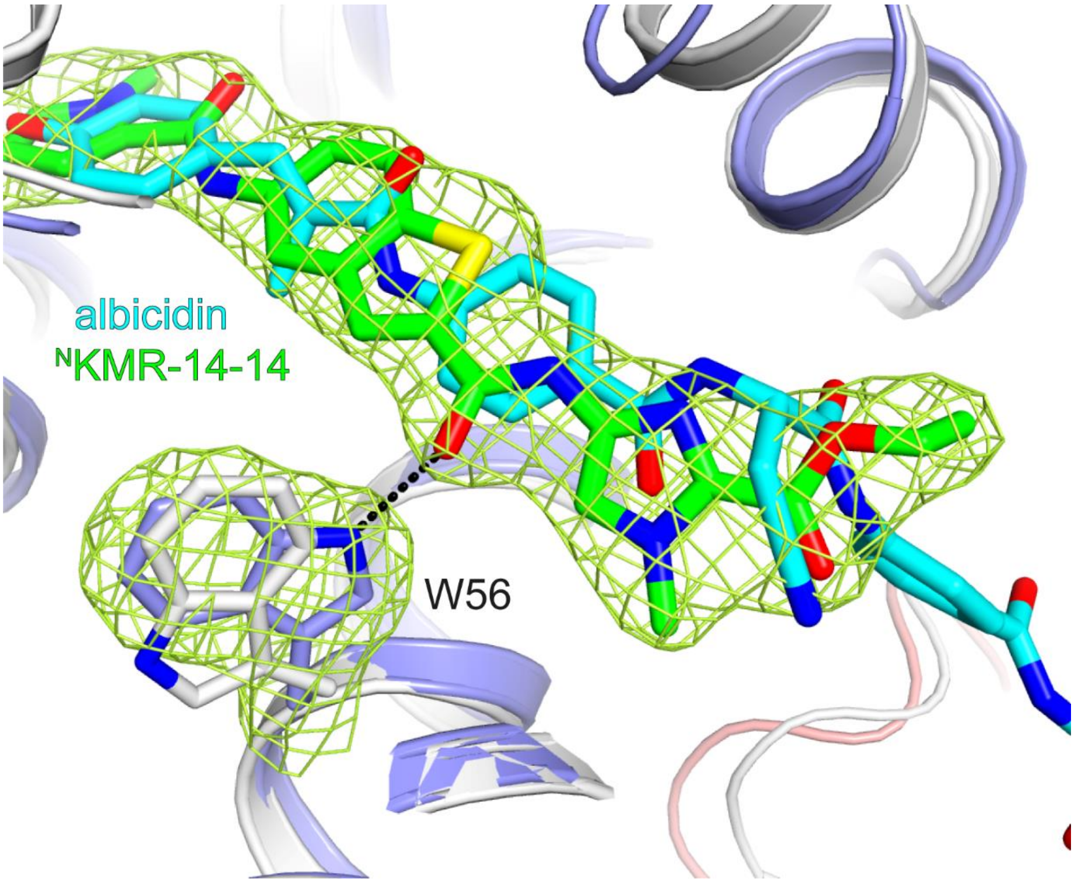

Supplementary Figure 3. Rotamer selection by ligand-specific interaction. A carbonyl group of KMR-14-14 is located where a benzene ring is found in the AlbAS:albicidin complex. The former is stabilised by a H-bond interaction (shown by the broken line) with the nitrogen atom of the indole group of W56. The sidechain of W56 is rotated in the AlbAS:albicidin complex allowing it to present the more hydrophobic portion of its side chain to the ligand.

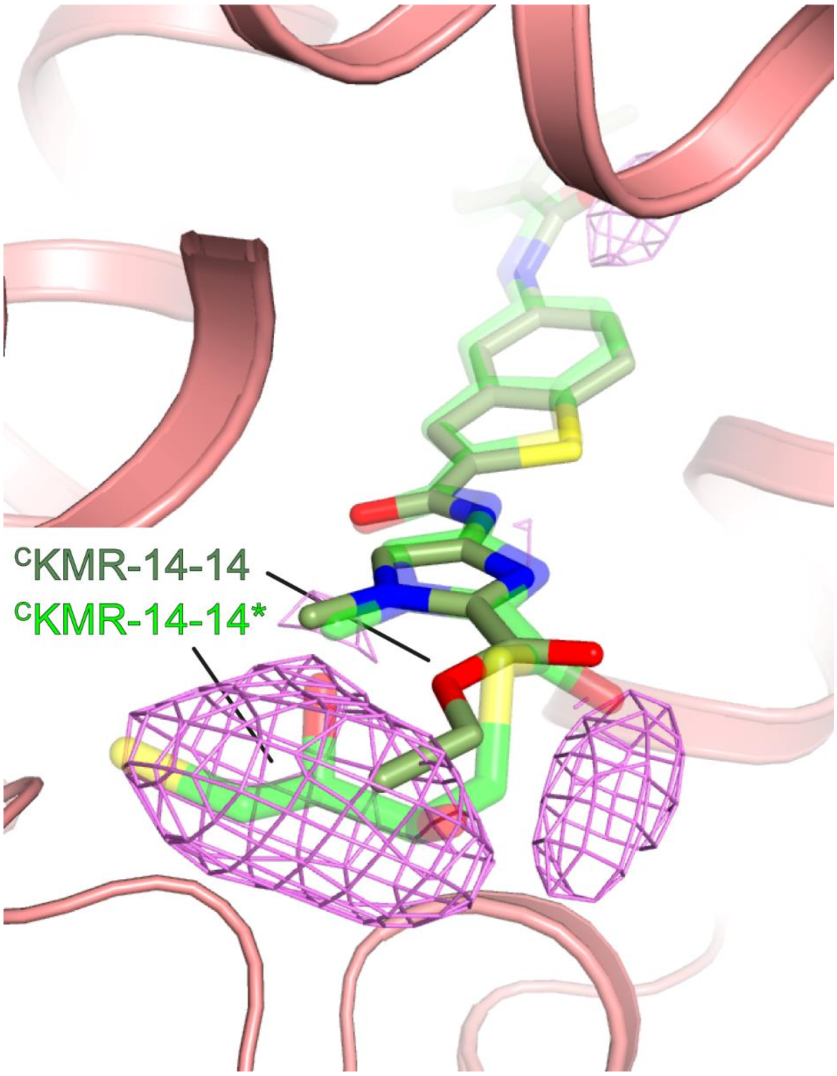

Supplementary Figure 4. KMR-14-14 can form a thioester adduct. In two out of three AlbAS molecules in the asymmetric unit, we find that refinement of KMR-14-14 bound at the CTD (^{C}KMR-14-14 shown in dark green) leaves a significant unaccounted difference in electron density at the ester tail (shown at the +4.0 σ level in violet). This can be explained by thioesterification following a reaction with a DTT molecule present in the buffer. The final refined thioester adduct (^{C}KMR-14-14*) is shown as partly transparent sticks in green for reference.

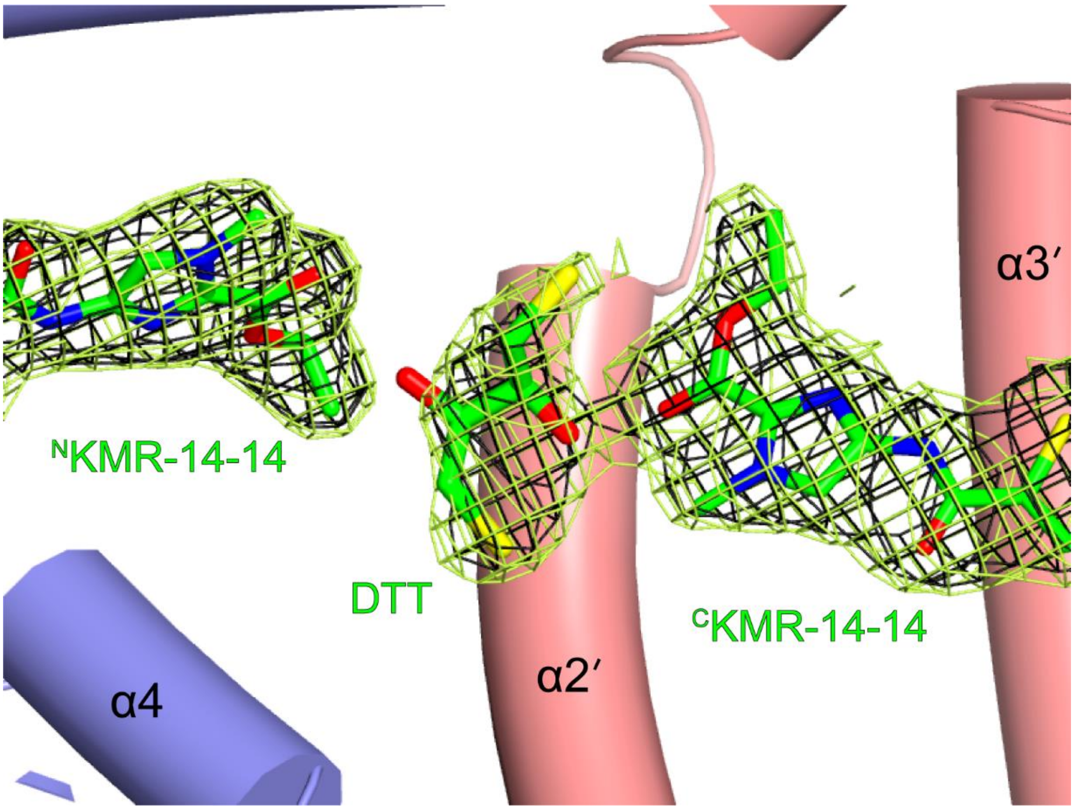

Supplementary Figure 5. A DTT molecule fills the gap between KMR-14-14 tails. In one of the three AlbAS molecules present in the asymmetric unit, a DTT molecule is found in proximity of ^{C}KMR-14-14. This arrangement is presumably the pre-reactive state resulting in the formation of the ^{C}KMR-14-14* thioester seen in the other AlbAS molecules (**Supplementary Figure 4**). 2mFo-DFc electron density for the ligand is shown at the 0.5 σ and 1.0 σ levels in light green and black respectively.

GGCGAGCGGCAATGAACCCGATCTGGAGTCCTGGCaACAGACGCTGGAGTTAATGAAAATGTACGATCGT

Supplementary Figure 6. The oligonucleotide used to generate the L120Q modification in the *albA* gene of *Klebsiella pneumoniae* NCTC 7427.

AlbAS species	Codon-optimised DNA sequence
Klebsiella oxytoca	GAAAACCTGTACTTCCAGGGTATGTACGACCGTTGGTTCTCTCAGCAGGAACTGCAGGTTCTGCCGTTCGCTGAACAGGACGAACAGCGTAACCAGACCTGGCTGGAACTGGTTGGTGAAGCTCAGCAGCTGATGGGTGAACGTTGCCCGGCTGACGAACCGCGTGCTATCGCTCTGGCTACCCGTTGGATGGAACAGCTGGAACAGGACACCGCTGGTCGTCCGGAATTCCTGACCCGTCTGAACGAAATGCACGCTGCTGAACCGCAGATGCGTGAACAGACCGGTGTTACCCCGGAAATGATCGACTTCATCACCCGTGCTTTCGCTGAATCTAAACTGGCTATCTGGGCTCGTTACCTGAACGCTGAAGAACTGGCTTTCACCCGTCAGCACTACTTCGACCGTCTGATGGAATGGCCGGCTCTGGTTGCTGACCTGCACCGTGCTTGCCGTGAAAAACGTGACCCGGCTTCTCCGGAAGGTCAGCAGCTGGCTCAGCGTTGGCTGGCTCTGTTCCAGTCTTACGCTGGTAAAGACGCTCAGACCCAGCAGAAATTCCGTTACGCTATGGAACAGGAACCGCACCTGATGAAAGGTACCTGGATGACCTCTGAAGTTCTGTCTTGGCTGCAGCAGGCTATCGGTGTTATGATGCGTCAGGCTCAGGGTCCGGCTGCTGAAGGATCCGTTTCTGGTTGGCGTCTGTTCAAGAAGATCTCTGGATCCTAG
Klebsiella pneumoniae	GAAAACCTGTACTTCCAGGGTATGTACGACCGTTGGTTCTCTCAGCAGGAACTGGCTGCTCTGCCGTTCGCTGCTCAGGACGAACAGCGTGCTCAGGCTTGGCGTGAACTGACCGAAGAAGTTCAGACCCTGATGGCTTCTGGTTGCCCGACCGACTCTCCGCAGGCTATGTCTCTGGCTACCCGTTGGATGGAACGTCTGGAACAGGACACCGCTGGTCGTCCGGAATTCCTGACCCGTCTGAACGCTATGCACGCTGCTGAACCGCAGATGGTTGAACAGACCGGTGTTACCCCGGCTATCATCGCTTTCATCACCGAAGCTTTCGCTGAATCTAAACTGGCTATCTGGGCTCGTTACCTGGACGACGAAGAAATGGCTTTCACCCGTCAGCACTACTTCGACCGTCTGCAGGAATGGCCGGCTCTGGTTGCTAAACTGCACCAGGCTTGCCGTGAAGGTATCGCTCCGGACTCTGCTTCTGGTCAGGCTCTGGCTCGTGCTTGGCTGGAACTGTTCCAGTCTTACGCTGGTACCCGTCCGCAGACCCTGCAGAAATTCCGTCGTGCTATGGAACAGGAACCGCACCTGATGAAAGGTACCTGGATGACCCCGGCTGTTCTGTCTTGGCTGCAGCAGGCTACCGGTGCTGTTATGCGTCAGGCTCAGGGTCCGGCTGCTGGTGGATCCGTTTCTGGTTGGCGTCTGTTCAAGAAGATCTCTGGATCCTAG

Supplementary Table 6. The sequences for the *Klebsiella oxytoca* and *Klebsiella pneumoniae albAS* genes used for Gibson assembly.

Name	Sequence
AlbAS_forward primer	CTGGTGCCGCGCGGCAGCCATATGGAAAACCTGTACTTCCAG
AlbAS_reverse primer	GTGCTCGAGTGCGGCCGCCTAGGATCCAGAGATCTTC
pET28a(+)_forward primer	GAAGATCTCTGGATCCTAGGCGGCCGCACTCGAGCAC
pET28a(+)_reverse primer	CTGGAAGTACAGGTTTTCCATATGGCTGCCGCGCGGCACCAG

Supplementary Table 7. The primers used for Gibson assembly of the *Klebsiella oxytoca* and *Klebsiella pneumoniae albAS* genes into the pET28a(+) vector.
